# Cisplatin resistance in gastric cancer cells is associated with HER2 upregulation-induced epithelial-mesenchymal transition

**DOI:** 10.1038/srep20502

**Published:** 2016-02-05

**Authors:** Dongsheng Huang, Hongying Duan, Hao Huang, Xiangmin Tong, Yong Han, Guoqing Ru, Like Qu, Chengchao Shou, Zhongsheng Zhao

**Affiliations:** 1 Clinical Research Institute, Zhejiang Provincial People’s Hospital, 158 Shangtang Road, Hangzhou 310014, China; 2 Department of Pathology, Zhejiang Provincial People’s Hospital, 158 Shangtang Road, Hangzhou 310014, China; 3 Key Laboratory of Carcinogenesis and Translational Research (Ministry of Education), Department of Biochemistry and Molecular Biology, Peking University Cancer Hospital & Institute, Beijing 100142, China

## Abstract

Cisplatin remains to be primary chemotherapeutic drug for gastric cancer patients, especially for advanced stage ones. However, primary or acquired resistance often occurs with the mechanisms being not well understood, which results in relapse of the cancer and poor survival. Herein, we found that HER2 upregulation was associated with cisplatin resistance. We observed that cisplatin-resistant gastric cancer cells underwent a morphological change similar to epithelial-mesenchymal transition (EMT) which is mediated by HER2 overexpression. When specific monoclonal antibody Herceptin, small molecular targeted drug CP724714, or small interfering RNA against HER2 was applied, the EMT-like phenotypic change was dramatically reversed. More importantly, the IC_50_ and Resistance Index of resistant gastric cancer cells to cisplatin were also decreased by any of these treatments.We demonstrated that expression and amplification of HER2 positively correlated with expression of EMT-related transcription factor Snail in gastric cancer tissues. Furthermore, for the first time, we found that HER2/Snail double positive gastric cancer patients had poorer survival than single positive or double negative counterparts, which provided experimental evidence for the necessity of HER2/Snail double testing in gastric cancer. In conclusion, this study provides some clues of the association of cisplatin resistance with HER2 upregulation-induced EMT in gastric cancer cells.

Gastric cancer is one of the most common malignancies worldwide. Despite recent advances in diagnosis and treatment as well as declining incidence in some developed countries, it remains a major cause of cancer-related deaths in China^1^,^2^. Gastric cancer in early stages is curable by using endoscopic procedures. However, it is difficult to cure in most advanced-stage patients and the clinical treatment options have evolved little^3^. Until now, chemotherapy still remains mainstream treatment for gastric cancer patients with advanced stage^4^,^5^,^6^. Among them, many are resistant to chemotherapy agents, including cisplatin^7^,^8^,^9^. So, investigating the mechanisms underlying chemotherapy-induced resistance has clinical significance.

Epithelial-mesenchymal transition (EMT) is a process in which epithelial cells lose their polarity and cell-cell adhesion signatures, and acquire the characteristics of mesenchymal cells, including spindle-cell shape, loss of polarity, intercellular separation, and pseudopodia formation^10^,^11^,^12^. During EMT, the expression of some epithelial cell markers, such as E-cadherin, Claudin1 and zonula occluden-1 (ZO1), decreased, while the expression of mesenchymal cell markers, such as vimentin and fibronectin, increased. EMT-related transcription factors, like Snail, Slug, ZEB1, ZEB2, and Twist, are also upregurated. EMT is involved in both physiological and pathological processes. It not only participates in embryonic development processes^10^, but also plays a critical role in many aspects of cancer biological behaviors, such as migration and invasion, metastasis and the acquisition of stem cell-like properties^12^,^13^,^14^,^15^. EMT of Cancer cells also associates with resistance to chemotherapy^16^,^17^,^18^,^19^. Up-regulation of Twist was associated with resistance to paclitaxel in human nasopharyngeal, bladder, ovarian, and prostate cancers^16^. In colorectal cancer, oxaliplatin-resistant cells can acquire the ability to migrate and invade with phenotypic changes resembling EMT^17^. In pancreatic and ovarian cancer, stable cell lines resistant to gemcitabine and paclitaxel established by continuous exposure can undergo EMT with increased expression of Snail and Twist^18^,^19^. However, there has no study investigating the role of EMT in mediating cisplatin resistance in gastric cancer.

HER2 (also known as ErbB2), a 185-kDa transmembrane tyrosine kinase (TK) receptor, is a member of the epidermal growth factor receptors (EGFRs) family. This family includes HER1 (also known as EGFR, ErbB1), HER2, HER3 (ErbB3), and HER4 (ErbB4). These receptors share similar molecular structure: an extracellular ligand-binding domain, a short transmembrane domain, and an intracellular domain with TK activity (excepting HER3)^20^. Unlike HER1, HER3 and HER4, HER2 has no ligand^20^. Recent studies indicate a role of HER2 in the development of many types of human cancer, especially in breast cancer^21^,^22^. HER2 overexpression and/or amplification have been detected in 10–34% of invasive breast cancers and correlate with unfavorable patient outcome^22^. Targeted therapeutic drugs, including monoclonal antibody Herceptin and small molecule tyrosine kinase inhibitors targeting HER2, are playing more and more important roles in breast cancer treatment^23^,^24^. HER2 overexpression and/or amplification have also been observed in colon^25^, bladder^26^, ovarian^27^, endometrial^28^, lung^29^, uterine cervix^30^, head and neck^31^, esophageal^32^, and gastric carcinomas^33^. However, up to date, there is no study investigating the role of HER2 in mediating cisplatin-resistance in gastric cancer.

Based on the above knowledge, we hypothesized that HER2 and EMT may be involved in cisplatin resistance in gastric cancer. Herein, we found that HER2 is overexpressed in cisplatin-resistant gastric cancer cell models MGC803/DDP and AGS/DDP. Meanwhile, MGC803/DDP and AGS/DDP cells exhibited EMT-like morphological changes, compared with parental gastric cancer cell lines MGC803 and AGS. At molecular level, the alteration of expression patterns of EMT-related protein markers also implied this phenomenon. More interestingly, EMT in the cisplatin-resistant gastric cancer cells could be abrogated by monoclonal antibody Herceptin, small molecular targeted drug CP724714, or small interfering RNAs (siRNAs) against HER2. Furthermore, we demonstrated that amplification of HER2 or expression of HER2 positively correlated with the expression of EMT-related transcription factor Snail in 382 cases of gastric cancer, and HER2/Snail double positive patients had poorer patient outcome compared with single positive or double negative group.

## Results

### Cisplatin-resistant gastric cancer cell models display higher motility

**Figure 1:**
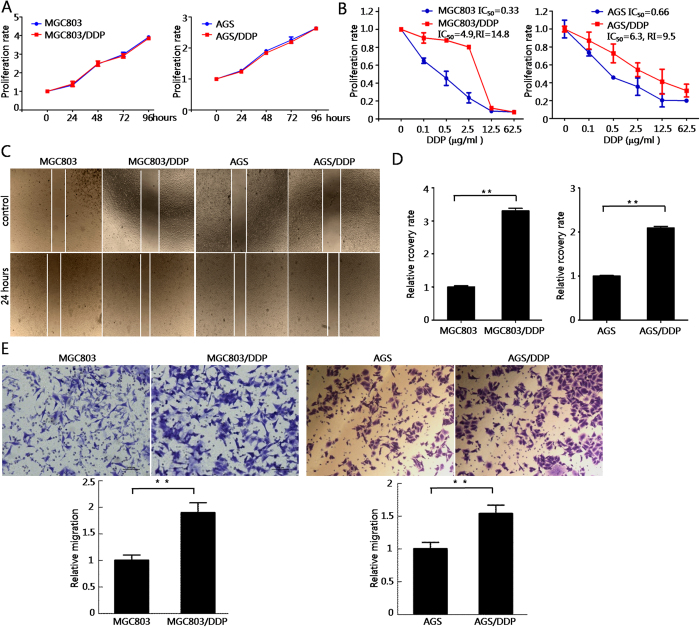
Cisplatin-resistant gastric cancer cell models display higher motility. (**A**) Cell growth curve of cisplatin-resistant MGC803 and AGS cells, versus parental MGC803 and AGS cells. Values represented the mean ± SD from two independent experiments with triplicate samples. DDP, cisplatin. (**B**) IC_50_ and RI for DDP in drug resistant and parental gastric cancer cells. Values represented the mean ± SD from three independent experiments with triplicate samples. RI, resistance index. (**C**) Cisplatin-resistant gastric cancer cells exhibit higher wound healing ability. Photos were taken at 0 and 24 h after wounding. (**D**) Summary of wound healing assays. Results were presented as the mean ± SD of two independent experiments, **P < 0.01 compared with parental cells. (**E**) Cisplatin-resistant gastric cancer cells possess higher cell migratory capacity *in vitro*. The results were mean ± SD of three independent experiments with triplicate wells. **P < 0.01.

First of all, we tested cell proliferation rate of cisplatin-resistant gastric cancer cells MGC803/DDP and AGS/DDP. As shown in Fig. 1A, the proliferation rates of parental and cisplatin-resistant cells were similar. Then, we tested the IC_50_ and the Resistance Index (RI) of MGC803/DDP and AGS/DDP. As shown in Fig. 1B, IC_50_ of MGC803/DDP and AGS/DDP cells were 4.9 and 6.3 μg/ml respectively, and the RI were 14.8 and 9.5 respectively. However, IC_50_ of MGC803 and AGS cells were 0.33 and 0.66 μg/ml respectively. Interestingly, we found that MGC803/DDP and AGS/DDP cells possess an increased motility compared with parental cells in the wound healing assay, as shown in Fig. 1C,D. Consistently, MGC803/DDP and AGS/DDP cells also showed enhanced migration through the micropore membranes into the lower chambers in the transwell chamber assay (Fig. 1E). The above results demonstrated that cisplatin-resistant gastric cancer cells displayed higher migratory capacity and motility compared with parental cells.

### Cisplatin-resistant gastric cancer cells exhibit an EMT-like phenotypic change

**Figure 2:**
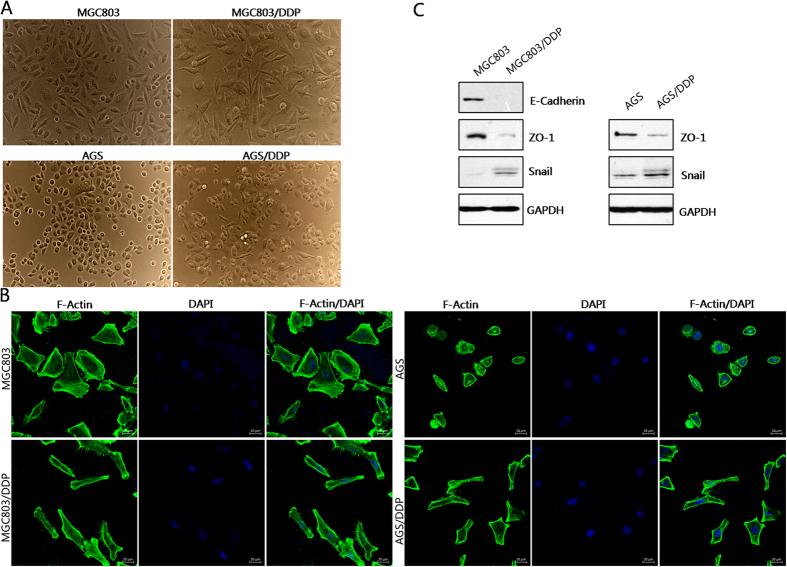
Cisplatin-resistant gastric cancer cells exhibit an EMT-like phenotypic change. (**A**) Representative images of cellular morphology of cisplatin-resistant MGC803/DDP and AGS/DDP cell lines, plus parental MGC803 and AGS cells. (**B**) FITC-phalloidin staining of F-actin in cisplatin-resistant MGC803/DDP and AGS/DDP cell lines, and parental MGC803 and AGS cells. (**C**) Epithelial marker E-cadherin, ZO1 and EMT-related transcription factor Snail were examined by Western blot.

EMT is of paramount importance in a plethora of cancer related events, including cancer metastasis and chemotherapy resistance^14^,^16^,^17^,^18^. But to the best of our knowledge, the association of EMT with cisplatin resistance in gastric cancer has not been reported. Herein, we found that MGC803/DDP and AGS/DDP cells exhibited a spindle-like fibroblastoid phenotypic change in contrast to parental cell lines MGC803 and AGS (Fig. 2A). FITC-conjugated phalloidin staining of F-actin also revealed that MGC803/DDP and AGS/DDP cells possessed EMT-like morphology compared with their parental cells (Fig. 2B). At molecular level, the expression patterns of EMT protein markers and EMT-related transcription factors were determined by western blot. MGC803/DDP and AGS/DDP cells showed reduced expression of epithelial cell marker ZO1. Meanwhile, the expression of E-cadherin was down-regulated in MGC803/DDP cells. Furthermore, the expression of EMT-related transcription factor Snail was increased in MGC803/DDP and AGS/DDP cells (Fig. 2C).

### HER2 is overexpressed and plays a pivotal role in mediating EMT in cisplatin-resistant gastric cancer cells

**Figure 3:**
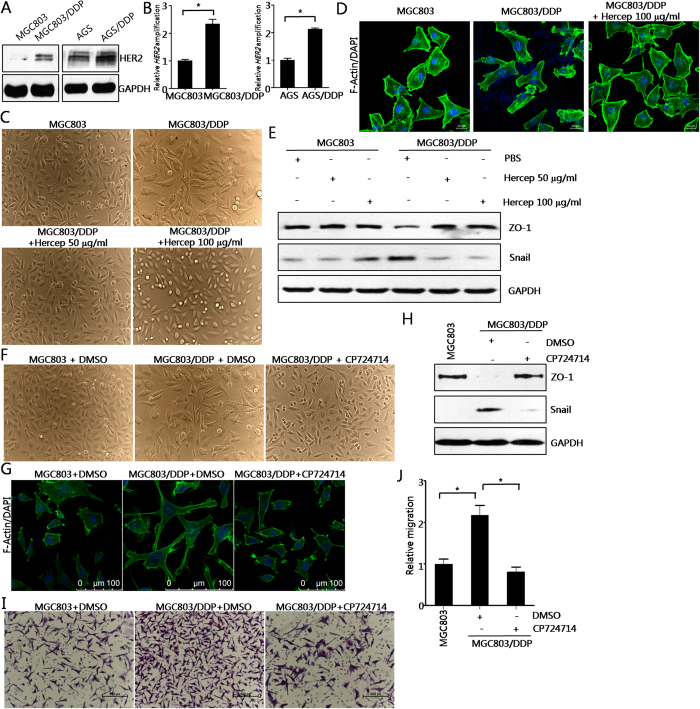
HER2 is overexpressed and both Herceptin incubation and siRNAs against HER2 reversed the EMT in cisplatin-resistant gastric cancer cells. (**A**) Western blot of HER2 in MGC803/DDP and AGS/DDP cell lines, and parental MGC803 and AGS cells. GAPDH was used as loading control. (**B**) Relative amplification of *HER2* in parental and cisplatin-resistant cells. Results were presented as the mean ± SD of two independent experiments, *P < 0.05 compared with parental cells. (**C**) Herceptin incubation abrogated EMT morphology in MGC803/DDP cells. Representative images of cellular morphology of MGC803/DDP cells, plus parental MGC803 cells treated with Herceptin for 24 h were shown. Hercep represents Herceptin. (**D**) Herceptin incubation reversed EMT morphology in MGC803/DDP cells. Representative images of FITC-phalloidin staining of F-actin after 100 μg/ml Herceptin treatment for 24 h were shown. (**E**) Effects of Herceptin on levels of ZO1 and Snail. (**F**) CP724714 treatment abolished EMT-like cell morphology. Cells were incubated with 10 μM CP724714 for 24 h. Representative images of cellular morphology of were captured. (**G**) CP724714 treatment abrogated EMT-like cell morphology. Cells were treated as in Fig. 4F, FITC-phalloidin staining of F-actin was performed and typical images were shown. (**H**) Effects of CP724714 on ZO1 and Snail. (**I**) CP724714 inhibited the increased cell migration in cisplatin-resistant gastric cancer cells. Representative images were presented here. (**J**) CP724714 inhibited the increased cell migration in cisplatin-resistant gastric cancer cells. Values represented the mean ± SD from three independent experiments with triplicate samples. *P < 0.01.

In the following study, we sought to explore the potential molecular mechanism which is responsible for the EMT-like phenotypic changes in cisplatin-resistant gastric cancer cells. Previous study revealed that HER2 overexpression and/or amplification is an unfavourable predictive factor^34^. So, we hypothesized that HER2 overexpression and/or amplification may be associated with cisplatin resistance in gastric cancer. As shown in Fig. 3A, HER2 expression was significantly upregulated in cisplatin-resistant gastric cancer cells, especially in MGC803/DDP cells. Next, we found that MGC803/DDP and AGS/DDP cells showed increased HER2 amplification compared with parental MGC803 and AGS cells through qPCR method, indicating that HER2 amplification played an important role in HER2 protein up-regulation (Fig. 3B).

In order to investigate the role of HER2 in mediating EMT in cisplatin-resistant gastric cancer cells, we utilized multiple strategies, including monoclonal antibody Herceptin blocking method, siRNA technique, and small molecular targeted therapeutic drug CP724714 treatment. Firstly, cell morphology analysis showed that the fibroblastic, spindle-shaped morphology of MGC803/DDP cells was reversed to the epithelial cell phenotype similar to that of parental counterparts after Herceptin treatment in a dose-dependent manner (Fig. 3C). Herceptin at the concentration of 100 μg/ml completely abrogated the EMT phenomenon observed in MGC803/DDP cells (Fig. 3C). Similar changes were observed by FITC-phalloidin staining for F-actin (Fig. 3D). At molecular level, ZO1 down-regulation and Snail up-regulation were also counteracted by Herceptin treatment in MGC803/DDP cells (Fig. 3E). Taken together, these results suggested that HER2 mediates EMT in cisplatin-resistant gastric cancer cells.

Next, a potent molecular targeted agent against HER2, CP724714, was utilized. CP724714 at the concentration of 10 μM was applied to MGC803/DDP cells for 24 h. As shown in Fig. 3F,G, the morphology of MGC803/DDP cells was also reversed to that of epithelial cells. Meanwhile, ZO1 down-regulation and Snail up-regulation were reversed by CP724714 in MGC803/DDP cells (Fig. 3H). CP724714 significantly lowered the cell migration of MGC803/DDP cells (Fig. 3I,J), suggesting that HER2 activity contributes to increased cell invasiveness in cisplatin-resistant gastric cancer cells.

**Figure 4:**
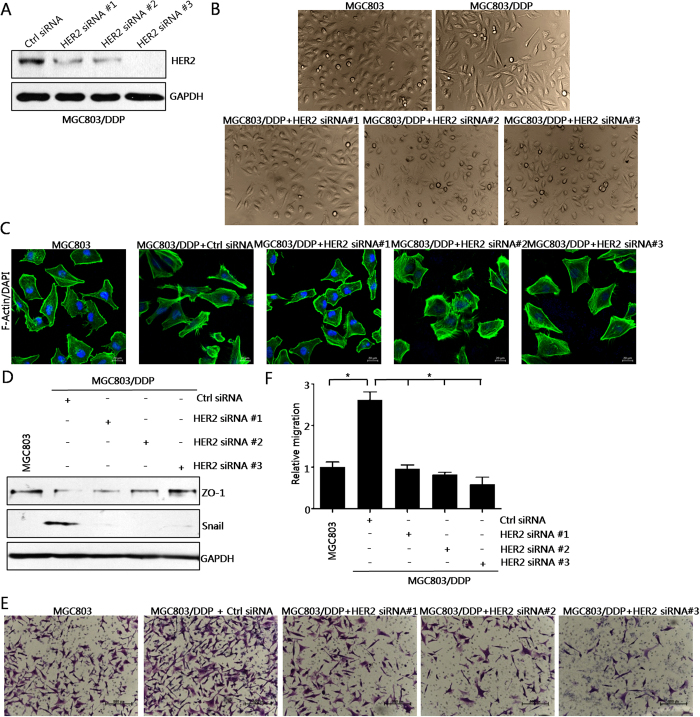
SiRNAs against HER2 could abrogate the EMT phenotype and cell invasiveness in cisplatin-resistant gastric cancer cells. (**A**) Validation of silencing efficiency of HER2. Cells were transiently transfected with 50 nM HER2-specific siRNAs #1-#3 or a control siRNA for 48 h. (**B**) Knock-down of HER2 abrogated EMT morphology. Cells were transiently transfected with 50 nM specific siRNAs targeting HER2 for 48 h, Representative images of cellular morphology were shown. (**C**) Knock-down of HER2 abolished EMT phenotype. Cells were treated as in Fig. 4B. FITC-phalloidin staining of F-actin were performed and representative images were shown. (**D**) Effects of HER2 knock-down on the levels of ZO1 and Snail. (**E**) Effects of HER2 knock-down on cell migration. Representative images of transwell cell migration assay were shown. (**F**) Summary of Fig. 4E. Values represented the mean ± SD from three independent experiments with triplicate samples. *P < 0.01.

Thirdly, we employed siRNA strategy. As shown in Fig. 4A, HER2 expressions were knocked down by three pairs of specific siRNAs. Cell morphology analysis and FITC-phalloidin staining assay showed that EMT of MGC803/DDP cells was completely reversed by these siRNAs (Fig. 4B,C). Snail up-regulation and ZO1 down-regulation were reversed by siRNAs in MGC803/DDP cells (Fig. 4D). Meanwhile, cell migration was inhibited by these siRNAs in MGC803/DDP cells (Fig. 4E,F).

Taken together, these data provided evidences that HER2 is required for EMT and cell invasiveness in cisplatin-resistant gastric cancer cells.

### HER2 inhibition significantly reverses cisplatin resistance in MGC803/DDP cells

**Figure 5:**
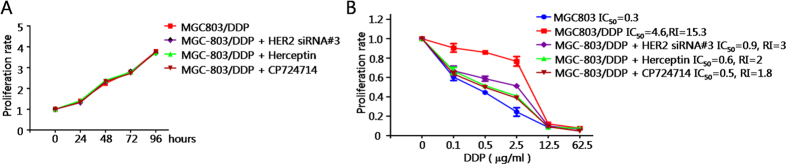
HER2 inhibition significantly reverses cisplatin resistance in MGC803/DDP cells. (**A**) Cell growth curve of cisplatin-resistant MGC803 cells with different treatments. Values represented the mean ± SD from two independent experiments with triplicate samples. (**B**) Effects of different treatments on IC_50_ and RI. Values represented the mean ± SD from three independent experiments with triplicate samples.

Firstly, we tested whether multiple HER2 inhibition strategies affect the proliferation of MGC803/DDP cells. After transfection with specific siRNA#3, treated with 100 μg/ml Herceptin, or treated with 10 μM CP724714, the proliferation rate remained similar (Fig. 5A). Next, we calculated the IC_50_ values and Resistance indexes of each panel. As shown in Fig. 5B, the sensitivity of MGC803/DDP cells towards cisplatin was dramatically increased (Fig. 5B). The IC_50_ values was decreased to 0.9, 0.6, or 0.5 respectively after siRNA against HER2, Herceptin, or CP724714 pre-treatment. These data suggested that HER2 is required for the maintenance of cisplatin resistance in gastric cancer cells. Meanwhile, it also provided potential therapeutic strategies for gastric cancer patients being unresponsive with cisplatin.

### HER2/Snail double positive gastric cancer patients have unfavorable outcome

**Figure 6:**
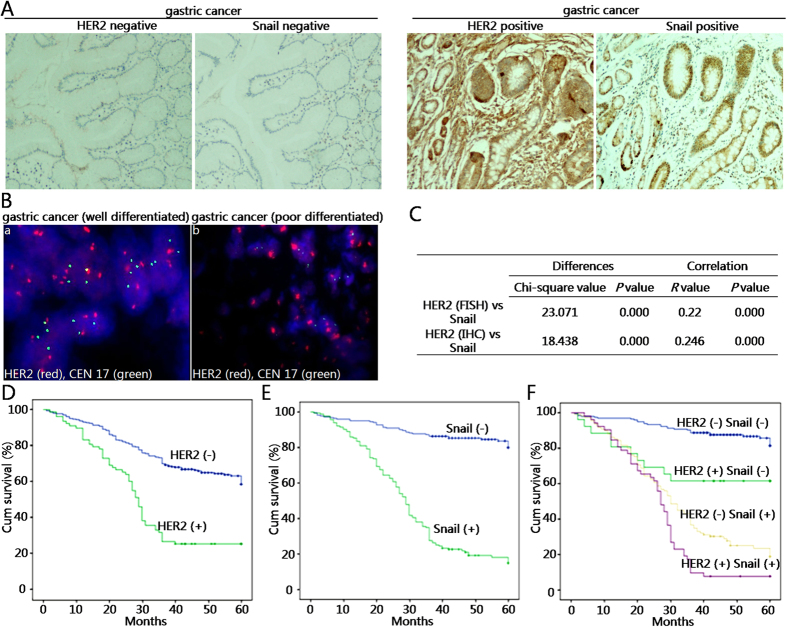
HER2/Snail double positive gastric cancer patients have unfavorable outcome. (**A**) Representative immunohistochemical staining of HER2 and Snail by respective antibody in human gastric tissues. Original magnification, 100 × . (**B**) Representative FISH staining of HER2 by probe in human gastric tissues. Original magnification, 400 × . HER2 amplification was determined as a ratio of HER2 (red dots) and chromosome 17 centromere (green dots) signal counts. Ratios < 2 were determined as no amplification with FISH, and those >2 as positive amplification with FISH. “a” represents no amplification; “b” represents positive amplification. (**C**) Chi-square analysis of the correlation of HER2 expression or amplification with Snail expression. (**D**) Kaplan-Meier Curve analysis of HER2 amplification with OS for patients with gastric cancer by the log-rank test. (**E**) Kaplan-Meier estimation of Snail overexpression with OS for patients with gastric cancer by the log-rank test. (**F**) Kaplan-Meier estimation of HER2/Snail double positive expression versus single positive or both negative counterparts with OS for patients with gastric cancer by the log-rank test.

To determine the association of HER2 overexpression and/or amplification with EMT in gastric cancer at tissue levels, we examined HER2 protein expression by immunohistochemistry (IHC) and DNA amplification by fluorescence *in situ* hybridization (FISH) in a cohort of 382 gastric cancer samples with a 5-year follow-up. Meanwhile, Snail protein expression was also tested by IHC in the same cohort. As shown in Fig. 6A, HER2 expression was positively correlated with Snail expression. In these cases, HER2 amplification was determined as a ratio of HER2 to that of chromosome 17 centromere signal. The typical results of the FISH test were presented in Fig. 6B.

In this cohort of gastric cancer patients, 19.9% (76/382) by IHC and 20.4% (78/382) by FISH were positive for HER2 (Table 1). 42.7% (109/382) by IHC were positive for Snail (Table 1). Although HER2 expression and/or amplification exhibited no link with patient’s gender, age, tumor size, or tumor differentiation, significant correlations with depth of invasion, lymph node metastasis, TNM staging, vascular invasion and metastasis were observed (Table 1). Snail expression obviously correlated with tumor size, differentiation, depth of invasion, lymph node metastasis, TNM staging, vascular invasion and metastasis (Table 1). Next, Chi-square statistical analysis was employed to evaluate the correlation of HER2 overexpression and/or amplification with Snail expression. The results showed that expression of Snail positively correlated with HER2 expression or HER2 amplification (Fig. 6C).

To analyze the correlation of HER2 expression/amplification and Snail expression with patients’ outcome, we performed patient survival analysis. The Kaplan-Meier plotting showed that gastric cancer patients with increased HER2 amplification had a poorer overall survival (OS) than those with HER2-negative counterparts (Fig. 6D). Gastric cancer patients with Snail-positive expression also had a poorer OS than those with Snail-negative expression (Fig. 6E). These two findings were consistent with previous reports by other labs^34^,^35^,^36^. Importantly, we found gastric cancer patients with HER2/Snail double positive amplification/expression had worst OS than those with single positive expression/amplification or both negative groups (Fig. 6F). These data indicated that combined testing of HER2 and Snail expression and/or amplification had a significantly prognostic value for determining the survival of gastric cancer patients.

## Discussion

Gastric cancer remains the second leading cause of cancer mortality in the world^37^. Surgical resection is the primary option of treatment and can cure patients with early-stage cancer. However, many gastric cancer patients are diagnosed when the tumor is at an unresectable stage. For these persons, application of chemotherapeutic agents singly or in combination remains mainstream treatment. Cisplatin, as a first-line chemotherapeutic drug for gastric cancer, could trigger apoptosis by inducing DNA damage through crosslinking of the DNA^38^. However, cancer cells often develop multiple mechanisms to overcome cisplatin-induced DNA damage and apoptosis, leading to cisplatin resistance^39^,^40^. Therefore, investigation of the molecular mechanism conferring chemotherapy resistance is urgently needed.

Although HER2 plays a povital role in gastric cancer progression and prognosis^34^, the molecular mechanisms of HER2 amplification and/or overexpression in mediating gastric cancer-related biological behaviors is not yet fully understood. To our knowledge, this is the first study investigating the association of HER2 with cisplatin resistance in gastric cancer cells.

Herein, we found that HER2 protein is overexpressed in cisplatin-resistant gastric cancer cells compared to parental cells, likely due to HER2 gene amplification. Moreover, we found that these cells underwent an EMT-like morphological alteration. EMT is associated with cancer aggressiveness, invasive and metastatic potential, and chemotherapeutic resistance^14^,^15^,^16^,^17^,^18^. In recent decade, the molecular mechanisms and signaling pathways mediating EMT were extensively studied, leading to the discovery of several pathways involved in the loss of epithelial cell polarity and the acquisition of mesenchymal phenotypic traits^41^,^42^,^43^. However, there is few study linking HER2 and EMT with cisplatin resistance in gastric cancer. In this study, we found that HER2 upregulation is responsible for EMT phenomenon and these events are associated with cisplatin resistance in gastric cancer cells. It was demonstrated that progression of carcinoma cells to metastatic tumor cells frequently involved EMT-like epithelial plasticity changes towards a migratory, fibroblastoid phenotype, particularly evident at the invasive front of human tumors^44^. Our current study suggests that EMT is not only a cause of cisplatin resistance in gastric cancer, it may also be a consequence of cisplatin resistance in gastric cancer which results in increased cancer metastasis and tumor relapse after cisplatin treatment for the unresponsive patients.

Herceptin is a monoclonal antibody which specifically targets HER2 protein by directly binding the extracellular domain of the receptor. Herceptin enhances survival rates in both primary and metastatic HER2-positive breast cancer patients^45^,^46^. The efficacy of Herceptin in breast cancer patients has led to its use in other HER2-positive cancer patients, including gastric cancer^47^,^48^,^49^. In this study, we found that gastric cancer patients with HER2 overexpression and/or amplification had a more unfavourable outcome. Kaplan Meier curve analysis demonstrated that Snail is another prognostic factor in gastric cancer progression, which is consistent with previous report^36^. Our study also suggested that combined testing of HER2 and Snail expression in gastric cancer would provide more useful information for patient prognosis, especially for those respond badly to cisplatin, however more efforts are required to validate this assumption. It is reported that HER2 is upregulated after onset of acquired resistance to cisplatin and fluorouracil combination chemotherapy in gastric cancer patients (*P* = 0.0065)^50^. The microarray data presented in this article implies that HER2 is upregulated in cisplatin-resistant gastric cancer samples. Furthermore, the mechanism of cisplatin resistance-induced HER2 overexpression in gastric cancer and the signaling pathway mediating HER2-upregulation-induced EMT in cisplatin-resistant gastric cancer cells also need to be elucidated in the future.

In conclusion, this study links HER2 overexpression-mediated EMT with cisplatin resistance in gastric cancer for the first time. Our study also suggests that targeting both HER2 and Snail would be a useful therapeutic strategy for the prevention of gastric cancer metastasis.

## Methods

### Cell culture

Human gastric cancer cell line MGC803 was kept in Peking University Cancer Hospital & Institute. Human gastric cancer cell line AGS was obtained from ATCC (American Type Culture Collection). MGC803 was from a 53-year-old Chinese male with poorly differentiated gastric mucinous adenocarcinoma^51^. AGS was from a 54-year-old Caucasian female with poorly differentiated gastric adenocarcinoma. The above two cell lines were cultured in RPMI-1640 medium supplemented with 10% fetal bovine serum (FBS) in 5% CO_2_ at 37 °C. The culture media and FBS were obtained from Invitrogen (Carlsbad, CA, US). Cisplatin was obtained from Sigma-Aldrich (St. Louis, MO, USA). The cisplatin-resistant MGC803/DDP cells were developed from the parental MGC803 cells that were subjected to gradient exposure to cisplatin as described in^52^. The cisplatin-resistant AGS/DDP cells were obtained by the same way.

### Reagents and antibodies

FITC-phalloidin (P5282) was purchased from Sigma-Aldrich, dissolved in methanol, aliquoted and stored at –20 °C according to manufacturer’s instructions. EMT antibody sampler kit (9782) was purchased from Cell Signaling (Danvers, MA, US). Monoclonal antibody against HER2 (ZM-0065) was purchased from Zhongshan Golden Bridge Biologicals (Beijing, China). Polyclonal antibody against Snail (NBP2-27293SS) was purchased from Novus Biologicals (Littleton, CO, US). Polyclonal antibody against ZO-1 (ab59720) was purchased from Abcam (Cambridge, MA, US). CP724714 (S1167) was purchased from Selleck Chem (Shanghai, China). 3-(4,5-cimethylthiazol-2-yl)-2,5-diphenyl tetrazolium bromide (MTT) and dimethyl sulphoxide (DMSO) were purchased from Sigma-Aldrich.

### Western blot

Cells were harvested with a plastic scraper and then washed twice with cold PBS. The cells were then homogenized in lysis buffer (25 mM Tris-HCl pH 7.4, 150 mM NaCl, 1 mM EDTA, 1% NP-40, 10% glycerol, and 1x complete protease inhibitors). The proteins of the lysates were quantified with BCA^TM^ Protein Assay Kit (Thermo). 50 μg of total proteins were subjected to SDS-PAGE and Western blot as described in^53^.

### Quantitative PCR (qPCR) analysis of HER2 amplification

qPCR was performed on an ABI7300 thermocycler (Life Technologies Corporation) by a previously described method^54^. Primers of *HER2* and IGF1 (synthesized by Shanghai Sangon) were as follows: *HER2* forward primer 5′-GAACTGGTGTATGCAGATTGC-3′, *HER2* reverse primer 5′-AGCAAGAGTCCCCATCCTA-3′, *IGF1* forward primer 5′-AGCTCGGCATAGTCTT-3′, *IGF1* reverse primer 5′-CCAAGTGAGGGGTGTGA-3′. The standard amplification protocol consists of an initial denaturation step at 95 °C for 10 min, followed by 50 amplification cycles at 95 °C for 10 sec, 55 °C for 5 sec, and 72 °C for 10 sec.

### Cell Mobility Assay

Cell mobility was assessed using a wound healing assay. The parental and cisplatin-resistant gastric cancer cells were seeded into six-well plates and cultured to monolayers, which were then wounded using sterile pipette tips and any cellular debris was removed by washing with PBS. The wounded monolayers were then incubated in medium containing 10% FBS for 24 h. Photos were captured at 0 and 24 h after wounding.

### Cell migration assay

Tissue culture-treated 6.5-mm Transwell chamber with 8.0-μm pore membranes (Corning, Corning, NY, US) was used in the cell migration assay. The assay was performed as described in^55^.

### Immunofluorescence

Cells were grown on the coverslips and fixed with 4% fresh paraformaldehyde for 10 min at room temperature, followed by blocking with 5% bovine serum albumin (BSA) at room temperature for 1 h. Next, cells were stained with FITC-Phalloidin for 1 h at room temperature in dark. After washing, cells were counterstained with DAPI for 10 min and mounted on 50% glycerol/PBS. A Leica SP2 confocal microscope (Leica Microsystems, Dresden, Germany) was used to observe the distribution of F-actin.

### SiRNA transfection

Cells were plated in 12-well plates and transfected with siRNA mate (GenePharma, Shanghai, China) according to the manufacturer’s protocol. The target sequences were as follows: HER2 #1 5′-ATCACAGGTTACCTATACA-3′, #2 5′-TGTCAGTATCCAGGCTTTGTA-3′, #3 5′-AAATTCCAGTGGCCATCAA-3′. Negative control siRNA sequence was 5′-ACACGAGAUAAUAUCGACUUG-3′. siRNAs were synthesized by GenePharma.

### The MTT cell proliferation assay

1 × 10^4^ cells were plated in 96-well plates before experiments. After HER2 siRNA transfection for 24 h, 100 μg/ml Herceptin pre-treatment for 2 h or 10 μM CP724714 pre-treatment for 2 h, different concentrations of cisplatin (0.1, 0.5, 2.5, 12.5 and 62.5 μg/ml) were applied for 72 h. Next, 10% MTT was added to the wells. After incubated at 37 °C for 4 h, the mixture reacted in culture media was centrifuged at 4000 rpm for 15 min. The precipitates were dissolved in DMSO. Absorbance was measured using a multiplate reader at the wavelength of 490 nm. Cell Proliferation Rate = Absorbance of drug treatment panel/Absorbance of control panel × 100%. Resistance Index = IC_50_ value of resistant cells/IC_50_ value of parental cells.

### Specimen and immunohistochemistry (IHC)

The study with clinical samples was approved by the Medical Ethic Committee of Zhejiang Provincial People’s Hospital. One cohort of formalin fixed and paraffin embedded (FFPE) gastric cancer tissues were obtained from archives of the Department of Pathology, Zhejiang Provincial People’s Hospital, which had 382 cases collected during 1998-2004. Written Informed Consents were obtained from all patients prior to operation. Specimens were diagnosed histopathologically and staged according to the TNM-International Union against Cancer classification system. IHC was performed to detect HER2 and Snail. The degree of immunostaining was reviewed and scored independently by two pathologist (Z.Z. and G.R.) based on the intensity of staining: 0 (no staining), 1 (weak staining = light yellow), 2 (moderate staining = yellow brown), and 3 (strong staining = brown). Moderate and strong stainings were defined as positive, while no and weak stainings were defined as negative.

### Fluorescence *in situ* hybridization (FISH)

FISH was performed on fixed-paraffin-embedded samples in the same cohort containing 382 gastric cancer cases. FISH experiments were performed according to the protocol given by the supplier (PathVysion kit, Vysis, Downers Grove, IL, US). The centromeric probe of chromosome 17 was included in FISH analyses in 382 cases. In these cases, HER2 amplification was determined as a ratio of HER2 and chromosome 17 centromere signal counts. Ratios ≤2 were determined as no amplification, and those >2 were defined as positive amplification

### Human rights statement and informed consent

The study with clinical samples was approved by the Medical Ethic Committee of Zhejiang Provincial People’s Hospital and was conducted in accordance with the Helsinki Declaration. Informed consent was obtained from all patients for being included in the study.

### Statistical analysis

Data were presented as mean ± SD. The differences were analyzed by ANOVA using SPSS 22.0 software and *P* < 0.05 was considered statistically significant. IC_50_ values were calculated using Graphpad Prism software.

## Additional Information

**How to cite this article**: Huang, D. *et al*. Cisplatin resistance in gastric cancer cells is associated with HER2 upregulation-induced epithelial-mesenchymal transition. *Sci. Rep.* **6**, 20502; doi: 10.1038/srep20502 (2016).

## Figures and Tables

**Table 1:** Correlations of HER2 or Snail expression with Clinicopathologic Factors.

Characteristics	No. of cases	HER2-positive-FISH (%)	HER2-positive-IHC (%)	Snail-positive(%)
Gender				
Male	272	51 (18.6)	50 (18.4)	109 (40.1)
Female	110	27 (24.5)	26 (23.6)	54 (49.1)
*p* value		0.203	0.244	0.107
Age (yrs)				
≤59	192	33 (17.2)	33 (17.2)	74 (38.5)
>59	190	45 (23.7)	43 (22.6)	89 (46.8)
*p* value		0.115	0.183	0.101
Size (cm)				
≤4	185	36 (19.5)	35 (18.9)	57 (30.8)
>4	197	42 (21.3)	41 (20.8)	106 (53.8)
*p* value		0.652	0.643	0
TNM stage				
I/II	163	15 (9.2)	15 (9.2)	24 (14.7)
III/IV	219	63 (28.8)	61 (27.9)	139 (63.5)
*p* value		0	0	0
Depth of invasion				
pT1 & pT2	138	18 (13.0)	19 (13.8)	24 (17.4)
pT3	244	60 (24.6)	57 (23.4)	139 (57.0)
*p* value		0.007	0.024	0
Differentiation				
WD & MD	109	25 (22.9)	25 (22.9)	34 (31.2)
PD	273	53 (19.4)	51 (18.7)	129 (47.3)
*p* value		0.441	0.347	0.004
LN metastasis				
Negative	138	13 (9.4)	13 (9.4)	23 (16.7)
Positive	244	65 (26.6)	63 (25.8)	140 (57.4)
*p* value		0	0	0
Vascular invasion				
Negative	156	13 (8.3)	13 (8.3)	26 (16.7)
Positive	226	65 (28.8)	63 (27.9)	137 (60.6)
*p* value		0	0	0
Metastasis				
Negative	324	59 (18.2)	57 (17.6)	117 (36.1)
Positive	58	19 (32.8)	19 (32.8)	46 (79.3)
*p* value		0.011	0.008	0
Positive Rate		20.4%	19.9%	42.7%
